# Mitogenomic analysis of a late Pleistocene jaguar from North America

**DOI:** 10.1093/jhered/esad082

**Published:** 2023-12-27

**Authors:** Megha Srigyan, Blaine W Schubert, Matthew Bushell, Sarah H D Santos, Henrique Vieira Figueiró, Samuel Sacco, Eduardo Eizirik, Beth Shapiro

**Affiliations:** Department of Ecology and Evolutionary Biology, University of California Santa Cruz, Santa Cruz, CA, United States; Department of Geosciences, Center of Excellence in Paleontology, East Tennessee State University, Johnson City, TN, United States; Department of Geosciences, Center of Excellence in Paleontology, East Tennessee State University, Johnson City, TN, United States; Department of Biology, University of Western Ontario, London, ON, Canada; School of Health and Life Sciences, Pontifical Catholic University of Rio Grande do Sul (PUCRS), Porto Alegre, RS, Brazil; School of Health and Life Sciences, Pontifical Catholic University of Rio Grande do Sul (PUCRS), Porto Alegre, RS, Brazil; Environmental Genomics Group, Vale Institute of Technology, Belem, PA, Brazil; Department of Ecology and Evolutionary Biology, University of California Santa Cruz, Santa Cruz, CA, United States; School of Health and Life Sciences, Pontifical Catholic University of Rio Grande do Sul (PUCRS), Porto Alegre, RS, Brazil; Department of Ecology and Evolutionary Biology, University of California Santa Cruz, Santa Cruz, CA, United States; Howard Hughes Medical Institute, University of California Santa Cruz, Santa Cruz, CA, United States

**Keywords:** ancient DNA, jaguar, mitochondrial DNA, Pleistocene

## Abstract

The jaguar (*Panthera onca*) is the largest living cat species native to the Americas and one of few large American carnivorans to have survived into the Holocene. However, the extent to which jaguar diversity declined during the end-Pleistocene extinction event remains unclear. For example, Pleistocene jaguar fossils from North America are notably larger than the average extant jaguar, leading to hypotheses that jaguars from this continent represent a now-extinct subspecies (*Panthera onca augusta*) or species (*Panthera augusta*). Here, we used a hybridization capture approach to recover an ancient mitochondrial genome from a large, late Pleistocene jaguar from Kingston Saltpeter Cave, Georgia, United States, which we sequenced to 26-fold coverage. We then estimated the evolutionary relationship between the ancient jaguar mitogenome and those from other extinct and living large felids, including multiple jaguars sampled across the species’ current range. The ancient mitogenome falls within the diversity of living jaguars. All sampled jaguar mitogenomes share a common mitochondrial ancestor ~400 thousand years ago, indicating that the lineage represented by the ancient specimen dispersed into North America from the south at least once during the late Pleistocene. While genomic data from additional and older specimens will continue to improve understanding of Pleistocene jaguar diversity in the Americas, our results suggest that this specimen falls within the variation of extant jaguars despite the relatively larger size and geographic location and does not represent a distinct taxon.

## Graphical Abstract

**Graphical Abstract:**
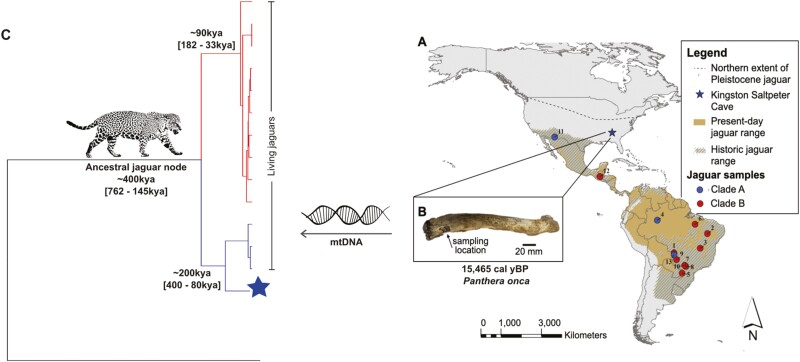

## Introduction

The jaguar, *Panthera onca*, is the largest living cat species native to the Americas and has a present-day distribution throughout much of South and Central America, extending into Mexico and the southern United States. Little is known, however, about the evolution and diversification of the jaguar lineage within the Americas. The jaguar lineage is believed to have evolved in Asia and dispersed into North America via the Bering Land Bridge at least once during the Pleistocene (Kurtén and Anderson 1980; Argant and Argant 2011; Li et al. 2016), after which it diversified as it spread throughout the Americas.

The jaguar fossil record is uneven temporally and geographically, with most published occurrences from the late Pleistocene of southern North America. In addition, size and proportional differences during the Pleistocene has led to uncertainties about the evolutionary history of this lineage in the Americas. The oldest known American fossil assigned to the jaguar lineage dates to 850 to 820 thousand years ago (kya) and is from the Hamilton Cave in West Virginia, United States (Seymour 1993). Further biochronological work by Martin et al. (2003) and Martin et al. (2009) suggests that the fauna from the section of Hamilton Cave where the jaguar remains were deposited could be as old as 1.3 to 1.6 million years ago (mya). Some authors suggest that the earliest occurrences of jaguar represent the extinct Eurasian/Palearctic species, *Panthera gombaszoegensis*, which is assumed to have crossed into Beringia ~1 mya, or a derived and distinct lineage of *P. onca* (Kurtén and Anderson 1980; Argant and Argant 2011). Furthermore, jaguar fossils in North America show size reduction in the late Pleistocene but are noted to be 15% to 20% larger than average-sized recent jaguars with differences in proportions (Kurtén and Anderson 1980; Seymour 1993). Living jaguars show high variation in body mass and usually weigh between 40 and 100 kg (Sunquist and Sunquist 2002; Hayward et al. 2016), whereas jaguar fossils from the Rancholabrean and Irvingtonian land mammal ages have been estimated to weigh between 85 and 120 kg (Seymour 1993). Based in part on these characteristics, some paleontologists assign North American jaguar fossils to a different species, *P. augusta* or subspecies *P. onca augusta* (Leidy 1872; Simpson 1941;McCrady et al. 1951; Kurtén and Anderson 1980; Schultz et al. 1985; Seymour 1993; Jiangzuo and Liu 2020). Similarly, fossil remains of large jaguars from Chile and Argentina have been assigned to *P. onca mesembrina*, which is considered endemic to Patagonia (Cabrera 1934; Martin 2018). However, these size estimates have occasionally been found to overlap with the largest living male jaguars reported, which weigh as much as 130 to 158 kg (Seymour 1989). Given this considerable morphometric variation in both fossil and living jaguars, the validity of these subspecific assignments remains unresolved.

Genomic analyses of fossil jaguars could determine whether Pleistocene jaguars were distinct from recent *P. onca* or simply represent temporal or geographic variation in body size, as well as improve understanding of the evolutionary history and diversification of this lineage. It is possible, for example, that jaguars became extinct locally in North America during the Pleistocene and later re-expanded from more tropical refugia (Eizirik et al. 2001; Lorenzana et al. 2022). This hypothesis emerges from genetic studies of present-day jaguars, which suggest repeated episodes of haplotype divergence and population expansion associated with sequential glacial–interglacial shifts in the late Pleistocene/early Holocene (e.g. Lorenzana et al. 2022). These climatic events may have driven jaguars to persist in refugia, resulting in regional extinctions and haplotype diversification over time. Genomic data from ancient specimens could be used to confirm or reject this hypothesis, as well as to better understand the evolutionary history, diversity, and taxonomy of jaguars in Pleistocene North America.

Here, we address this by extracting DNA from a large late Pleistocene jaguar fossil from Georgia, United States. We sequence and assemble a mitochondrial genome from this fossil and compare it to mitochondrial genomes from other living and extinct large cats. We find that this individual does not represent a separate Pleistocene lineage but instead falls within the extant diversity of jaguar mitochondrial lineages in South America. Our results confirm that jaguars dispersed northward into North America at least once during the Pleistocene and highlight the challenges of assigning phylogenetically meaningful taxonomy based on size or geographic location.

## Methods

### Sampling and radiocarbon dating

**Fig. 1.**
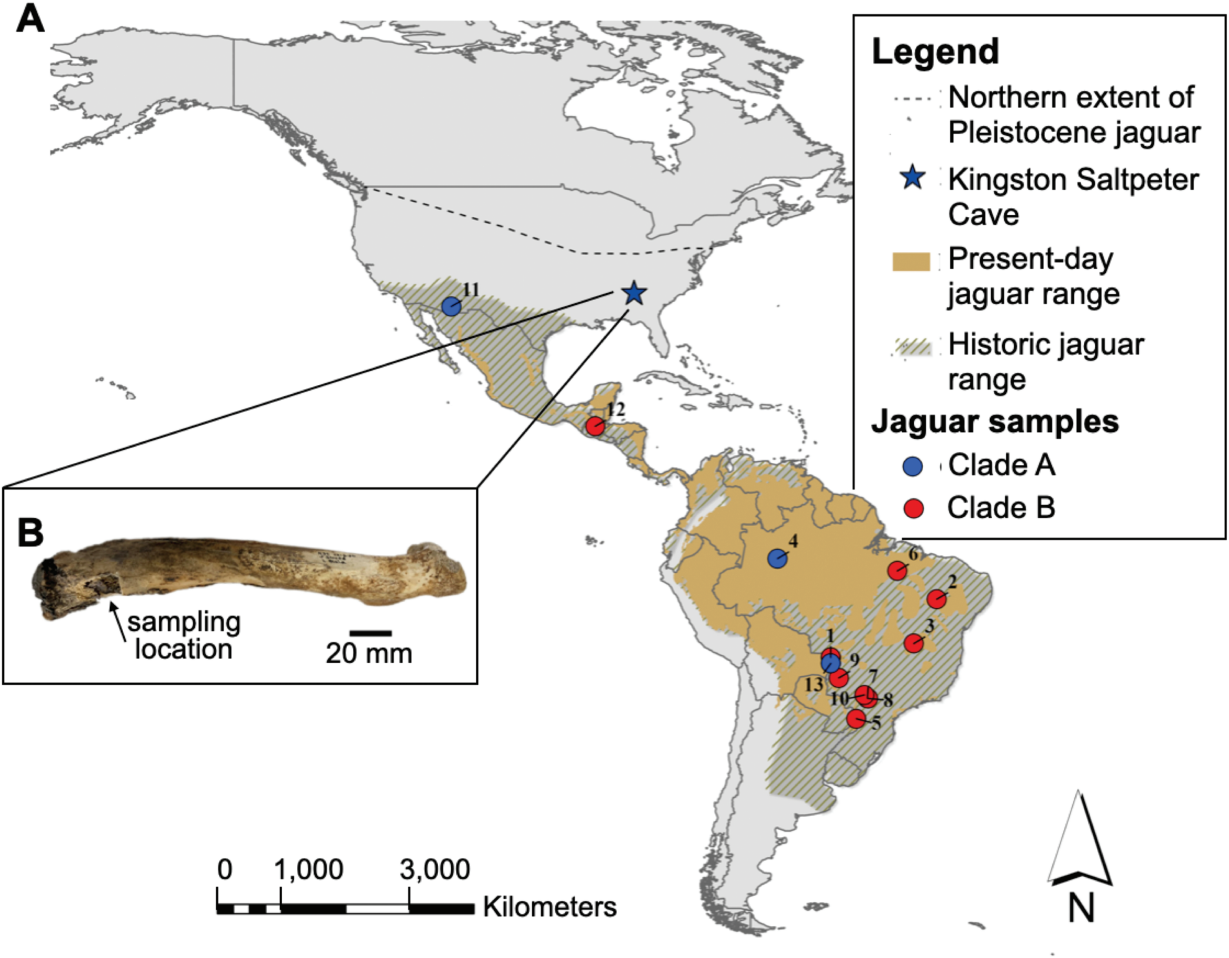
A) Map showing Pleistocene, historic (pre-1900), and present-day distribution of jaguars. Coloured circles show locations of recent jaguar samples. Clade notation and numbers refers to the phylogeny in Fig. 2. B) A photograph of the sampled Pleistocene jaguar bone. Left radius of *Panthera onca* from Kingston Saltpeter Cave, Georgia, United States. Arrow points to the location where the specimen was sampled for radiocarbon dating and aDNA analysis. Historical distribution of jaguars modified from Eizirik et al. (2001). Modern distribution of jaguar from Panthera (2017).

Fossil-bearing deposits in Kingston Saltpeter Cave, located in Bartow County, Georgia, in the Valley and Ridge province of the southern Appalachians, have yielded the remains of 41 mammalian taxa, including five extinct species (Fay and Wilson 2005). Radiocarbon dates on white-tailed deer (*Odocoileus virginianus*; 12,470 ± 50 14C yr BP) and long-nosed peccary (*Mylohyus nasutus*; 12,790 ± 50 14C yr BP) suggests a late Pleistocene age for the fauna (Sneed and Blair 2005). The Kingston Saltpeter jaguar material (Fig. 1) was identified as *P. onca augusta* by Fay and Wilson (2005) and includes 1 radius, 2 metacarpals, 1 calcaneus, 1 metatarsal, and 8 phalanges (Fay and Wilson 2005: p. 38).

We sent a subsample of the Kingston Saltpeter jaguar radius to Keck Carbon Cycle Accelerator Mass Spectrometry (AMS) Facility, University of California Irvine, for dating. The sample was decalcified in 1 N HCl, gelatinized at 60 °C and pH 2, and ultrafiltered to select a high molecular weight fraction, resulting in an AMS date of 12,940 ± 35 ^14^C yr BP (UCIAMS 259073; Schubert et al. in prep). We calibrated the date using OxCal (Bronk Ramsey 2009) assuming the IntCal20 calibration curve (Reimer et al. 2020), resulting in a 95.4% probability that the calibrated age is 15,630 to 15,300 cal BP (calibrated years before present, with present set as 1950). The sample is currently in the McClung Museum collection at the University of Tennessee, Knoxville, where it is cataloged as 2001.10.421. Prior to being at the McClung Museum, this specimen was housed at Berry College, Mount Berry, Georgia, where it was labeled as BC 83 (see Fay and Wilson 2005: p. 38).

### DNA extraction, library preparation, and hybridization capture

We collected bone powder from the Kingston Saltpeter jaguar radius that was sent for radiocarbon dating. To minimize the potential of contamination, we extracted DNA and prepared genomic libraries in a purpose-built ancient DNA facility at the UCSC Paleogenomics Lab using protocols designed for working with poorly preserved DNA (Fulton and Shapiro 2019). Prior to sampling, we decontaminated instruments and surfaces using bleach and ethanol. We subsampled the radius bone with a Dremel tool with a diamond saw attachment and powdered the removed bone fragment using a ball mill. We then aliquoted 50 mg of this powder and treated it with 0.05% bleach solution in a rotor for 15 min at room temperature followed by three subsequent spin and wash steps using molecular-grade water. We extracted DNA from this powder following a protocol for degraded DNA (Rohland et al. 2018) and used 1.5 ng as input for single-stranded libraries (Kapp et al. 2021). We enriched libraries for mitochondrial DNA using a custom bait set from Arbor Biosciences (Slon et al. 2016) enhanced with additional mammalian taxa (Kirillova et al. 2017). We used approximately 300 ng of library for enrichment, and followed the Arbor MyBaits protocol 5.0 with a hybridization and wash temperature of 60 °C. We sequenced the enriched library on a NextSeq 550 instrument using a 75 bp paired-end run with V3 chemistry, generating approximately 7 million read pairs.

### Mitochondrial genome assembly

To assemble a mitochondrial genome for the Kingston Saltpeter jaguar, we trimmed adapters, discarded reads with quality scores below 15, and merged reads with a minimum length of 30 bp and a minimum overlap of 15 bp using SeqPrep 2.0 (https://github.com/jstjohn/SeqPrep). To verify ancient DNA authenticity, we ran mapDamage2.0 (Jónsson et al. 2013). We filtered out low complexity sequences from the merged file using PRINSEQ (Schmieder and Edwards 2011), using the dust complexity method, and a complexity threshold of 7. This method searches for simple nucleotide repeats and assigns a complexity score based on the occurrence of these repeats in the data. We also used the flag -derep 124 to remove exact duplicates, 5ʹ duplicates, and reverse complement duplicates. Furthermore, since nuclear mitochondrial inserts (numts) are common in cats (Lopez et al. 1994, Kim et al. 2006), we adopted an assembly and basecalling approach that aimed to minimize their potential impact on our assemblies. We aligned reads simultaneously to the reference mitogenome and a well-characterized ~12.5 kb numt in *Panthera* (NCBI accession number DQ151551.1, Kim et al. 2006) using BWA-aln (Li and Durbin 2009) with parameters -l 16500 -n 0.01 -o 2 for the Kingston Saltpeter jaguar. Both alignments were sorted and filtered to remove unmapped reads using SAMtools (Li et al. 2009). We then used NumtParser (De Flamingh et al. 2023) to identify putative “numt” and mitochondrial ‘cymt’ reads in the data. For the ancient individual, we excluded 1,122 reads which were identified as numts. We then mapped the remaining complexity-filtered, deduplicated, numt-filtered, merged reads to the reference mitochondrial genome of *P. onca* (Genbank Accession #KM236783.1) using the short-read iterative assembler MIA (https://github.com/udo-stenzel/mapping-iterative-assembler). We did not explicitly filter for mapping quality scores, as the algorithm implemented in MIA uses a dynamic alignment scoring scheme based on read length in addition to a k-mer filter. We used an ancient substitution matrix with the alignment parameters -c -i -F and filter parameters -C -U -s -k 14, resulting in an average coverage of 26×. We called variants from the assembly output (.maln) file using a conservative approach to minimize ancient DNA-related variant calling bias. We used a minimum site coverage of 3× and MIA’s default consensus calling code (=1), which calls variants with an agreement of >65% across reads, to generate the consensus fasta file.

We assembled mitochondrial genomes from 12 recent jaguars using previously published shotgun sequence data (Figueiró et al. 2017; Lorenzana et al. 2022) (Table 1). After downloading the raw data, we trimmed the forward reads of each sample using Trimmomatic v.0.39 (phred score <20; sequence length <50; Bolger et al. 2014). As whole-genome shotgun data may include numt contamination, we generated alignments to the reference mitogenome and the 12.5-kb numt as above, using BWA-mem (Li 2013), followed by sorting, indexing, and removing unmapped reads using SAMtools (Li et al. 2009). We then used NumtParser (De Flamingh et al. 2023) and retained only those reads identified as mitochondrial (“cymt”) as input for the iterative assembler MIA, which were then employed to generate consensus assemblies using the jaguar mitogenome as the starting reference (NCBI accession no. KM236783.1). We called variants using mapping quality of 40, minimum coverage of 3×, and a consensus of at least 75% across reads. We then manually checked each assembly for premature stop codons, which might indicate the incorporation of numt sequences.

**Table 1.** Metadata for all individuals used in this study. Numbers following the common name of jaguars correspond to tree tips in Fig. 2.

Species	GenBank accession	Locality	Ref.
*Panthera onca* (Pleistocene Jaguar)	OR327006	Kingston Saltpeter Cave, Georgia, United States	This study
*P. onca* (Jaguar 1)	OR863188	Taiama reserve, North Pantanal, Brazil	Lorenzana et al. (2022)
*P. onca* (Jaguar 2)	OR863189	Serra da Capivara Natl. Park, Caatinga, Brazil	Lorenzana et al. (2022)
*P. onca* (Jaguar 3)	OR863190	Grande Sertão Veredas Natl. Park, Cerrado, Brazil	Lorenzana et al. (2022)
*P. onca* (Jaguar 4)	OR863187	Mamiraua reserve, Amazonia, Brazil	Lorenzana et al. (2022)
*P. onca* (Jaguar 5)	OR863191	Iguacu National Park, Atlantic Forest, Brazil	Lorenzana et al. (2022)
*P. onca* (Jaguar 6)	OR863192	Rondon do Para, Amazonia, Brazil	Lorenzana et al. (2022)
*P. onca* (Jaguar 7)	OR863193	Morro do Diabo State Park, Atlantic Forest, Brazil	Lorenzana et al. (2022)
*P. onca* (Jaguar 8)	OR863194	Morro do Diabo State Park, Atlantic Forest, Brazil	Lorenzana et al. (2022)
*P. onca* (Jaguar 9)	OR863195	Fazenda Caiman, South Pantanal, Brazil	Lorenzana et al. (2022)
*P. onca* (Jaguar 10)	OR863196	Porto Primavera region, Atlantic Forest, Brazil	Lorenzana et al. (2022)
*P. onca* (Jaguar 11)	OR863186	Madrean Woodland, Southern Arizona, United States	Lorenzana et al. (2022)
*P. onca* (Jaguar 12)	OR863197	Mayan Forest, Northern Guatemala	Lorenzana et al. (2022)
*P. onca* (Jaguar)	KM236783.1	Southern Pantanal, Brazil	Figueiró et al. (2017)
*P. leo* (Lion)	NC_028302.1	NA	Li et al. (2016)
*P. tigris* (Tiger)	NC_010642.1	NA	Lei et al. (2011)
*P. pardus* (Leopard)	NC_010641.1	NA	Lei et al. (2011)
*P. uncia* (Snow leopard)	NC_010638.1	NA	Lei et al. (2011)
*P. atrox* (American cave lion)	OK512983.1	Sixty Mile, Loc. 3, Yukon, Canada	Salis et al. (2022)

To compile a mitochondrial genome data set for phylogenetic analysis, we downloaded previously published mitochondrial genome sequences for lion, leopard, snow leopard, tiger, and the ancient lion *P. atrox* (Salis et al. 2022) (Table 1). We archived the newly assembled ancient mitogenome with the accession number OR327006 and recent jaguar mitochondrial genomes on NCBI with accession numbers OR863186 to OR863197.

### Alignment and phylogenetic analyses

We aligned the mitochondrial assembly of the Kingston Saltpeter jaguar, 12 recent jaguars, and the reference jaguar KM236783.1 to the mitochondrial genomes of the lion, leopard, tiger, snow leopard, and *P. atrox*, using tiger as the outgroup. We aligned the sequences using the ClustalW algorithm with 5 HMM iterations implemented in Geneious Prime 2023.1.2 (https://www.geneious.com) and manually inspected the alignment. We constructed a maximum-likelihood tree using RaxML v8.2.12 (Stamatakis 2014) with a GTR-GAMMA model of substitution, 100 bootstrap replicates, and a random starting seed and visualized it using FigTree v.1.4.4 (http://tree.bio.ed.ac.uk/software/figtree/).

We used the Bayesian approach implemented in BEAST v1.10.4 (Suchard et al. 2018) to estimate the timing of diversification among jaguar mitochondrial genomes. We partitioned the alignment generated above into coding regions, D-loop, and non-coding regions, as suggested by PartitionFinder2.0 (Lanfear et al. 2016), which identified the best substitution models to be GTR + G + I for all partitions. We compared models that assumed strict and relaxed clocks on the different partitions and assigned an uncorrelated relaxed clock for all partitions after inspecting the posterior distribution of the ucld.stdev parameter, which indicated non-zero rate variation across lineages (Supplementary Table 1). We assumed a constant population size for all partitions, and calibrated the molecular clock by assigning the radiocarbon age (15,465 ± 165 cal yBP) as a tip date for the Kingston Saltpeter jaguar, a previously estimated age for the ancient American lion, *P. atrox*, of 66,700 yr (Salis et al. 2022), which we sampled from a prior lognormal distribution with a mean of 66,700 yr (SD 10,000 and 95% CI 84,300 to 51,600 kya), and the previously estimated age of the common ancestor of *Panthera* of 3.72 mya (sampled from a lognormal distribution with SD 1,000,000.0 including 5.54 to 2.32 mya) (Johnson et al. 2006). Posterior distributions of parameters were estimated using MCMC sampling, with a total chain length of 100 million iterations and samples drawn every 10,000 steps. The first 10% samples were discarded as burn-in. Runs were assessed for effective sample sizes of parameters which were all >200. We performed two independent runs and checked for convergence using Tracer (Rambaut et al. 2018). We combined the tree files from both runs in LogCombiner and calculated the maximum clade credibility tree (MCC) in TreeAnnotator (Drummond and Rambaut 2007). We visualized the tree using FigTree v1.4.4 (http://tree.bio.ed.ac.uk/software/figtree/).

## Results

**Fig. 2.**
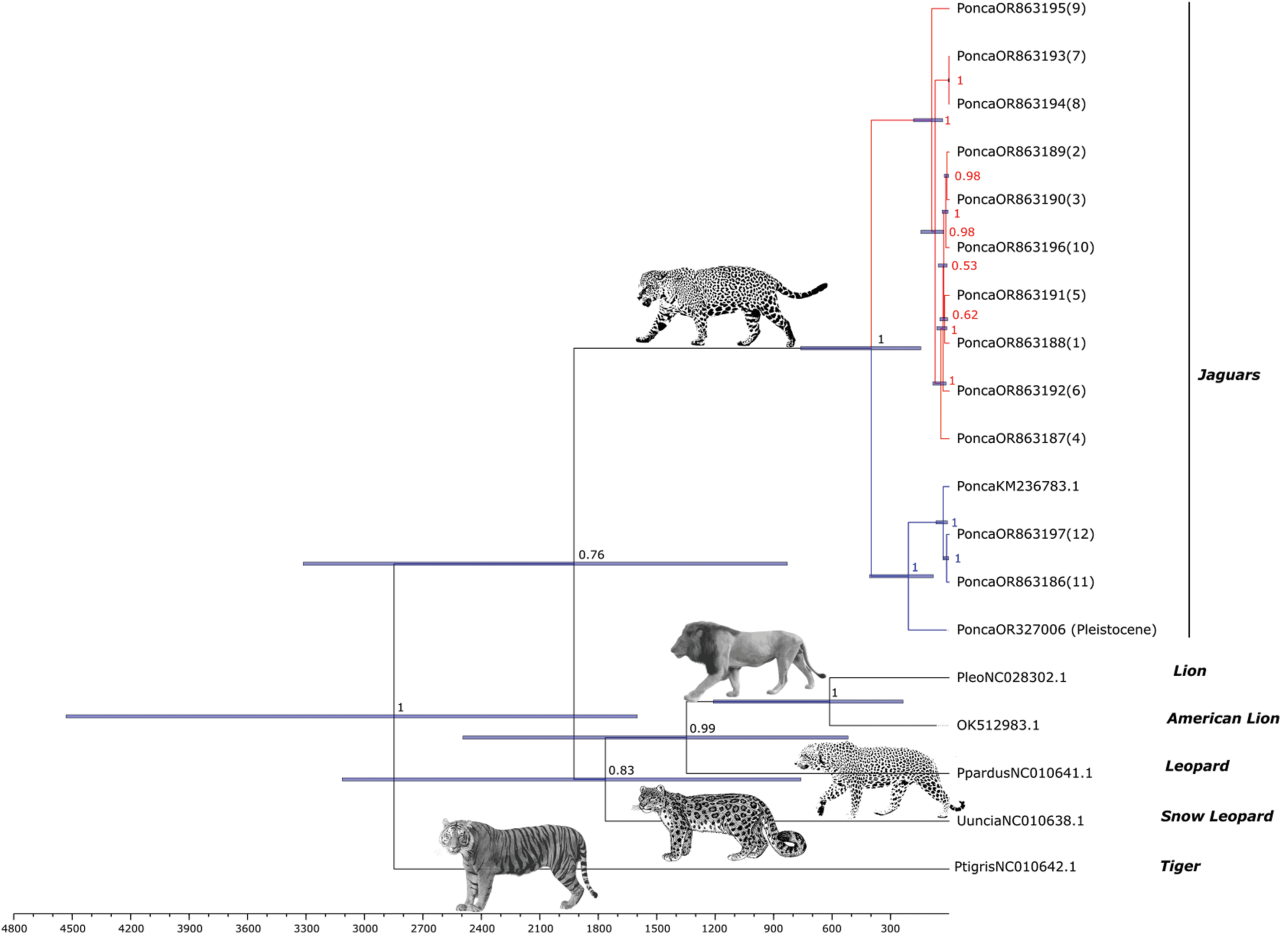
A time-calibrated maximum clade credibility tree of the ancient Pleistocene jaguar with recent jaguars and other members of *Panthera* including the ancient American lion *P. atrox*. The tree was generated using Bayesian analysis of whole mitochondrial genomes. Node values represent Bayesian posterior probabilities and tip labels for jaguars correspond to samples in the map in Fig. 1 and Table 1. Bars represent 95% highest posterior probability density intervals for node heights. All images sourced and modified from public domain (images of lion, leopard, snow leopard, and jaguar from Wikimedia Commons; image for tiger modified from the Smithsonian Institute’s public domain images).

We assembled a nearly complete mitochondrial genome from a late Pleistocene North American fossil jaguar from Kingston Saltpeter Cave, Georgia, United States, with an average of 26-fold coverage. This assembly has 16,658 base pairs (bp) and 405 missing bases (marked as “N”), covering 97.6% of the 17,063 bp reference mitogenome. The data exhibited elevated rates of C to T damage at the ends of the molecules, consistent with post-mortem damage patterns expected for ancient DNA. We aligned this ancient mitochondrial genome to mitochondrial genomes from 13 recent jaguars and performed phylogenetic analyses to infer the taxonomic relationship of the ancient jaguar to recent jaguars and other large cats in the *Panthera* genus. We estimated that the common ancestor of recent jaguars lived ~400 kya (Median age: 399 kya; 95% highest posterior density [HPD] 762 to 145 kya). This ancestral mitochondrial lineage split into two subclades, one of which (containing the Kingston Saltpeter jaguar) diversified ~209 kya (Median age: 209 kya, [95% HPD: 407 to 81 kya), and the other diversified much more recently ~90 kya (Median age: 88 kya, 95% HPD: 182 to 33 kya) (Fig. 2). Posterior estimates of the age of the *P. atrox* mitochondrial genome were consistent with the assigned priors, while the root of *Panthera* was estimated to be slightly younger (2.9 mya, 95% HPD 4.5 to 1.6 mya) but partially contained within the range estimated using nuclear data (Median age: 3.7 mya, 95% HPD 5.8 to 2.4 mya, Johnson et al. 2006). Posterior distributions of substitution rates for individual partitions are provided in Supplementary Table 2.

## Discussion

The mitochondrial genome of the Kingston Saltpeter jaguar from Georgia, United States, falls within the mitochondrial variation of recent jaguars. While we only have genetic data from a single individual from late Pleistocene North America, our results nonetheless confirm that temporal location and size differences between late Pleistocene and recent jaguars are not necessarily taxonomically informative. Jaguar fossils from the middle Pleistocene are larger than those from the late Pleistocene, the latter of which are 15% to 20% larger than average-sized extant jaguars (Kurtén and Anderson 1980; Seymour 1993). While the ecological or evolutionary changes leading to this progressive reduction in size are unknown, shortened limbs in Holocene jaguars may reflect a transition from a generalist to a more specialized predatory lifestyle (Kurtén and Anderson 1980).

Recent jaguars and the Kingston Saltpeter jaguar share a common ancestor during the Middle Pleistocene, 762 to 145 kya, around the time of the oldest jaguar fossils in North America. Jaguars later diversified into two main mitochondrial lineages. While the confidence intervals surrounding our estimates of diversification of these two subclades are wide, they overlap with previous estimates of diversification in jaguars from both mitochondrial and microsatellite data (Eizirik et al. 2001; Ruiz-García et al. 2013). We estimated that modern jaguar mitochondrial lineages diversified ~400 kya, similarly, to previous estimates of a mitochondrial haplotype expansion (Ruiz-García et al. 2013), although more sampling from older Pleistocene specimens could result in an earlier estimate for these clades. The median age of this timing, ~399 kya, follows the start of an interglacial Marine Isotope Stage (MIS, see Cohen and Gibbard 2019), MIS 11 (424 to 374 kya). Intriguingly, Lorenzana et al. (2022) inferred a signal of population decline for jaguars ~500 kya during an older interglacial event, MIS 13, using a PSMC approach with whole-genome data. Together, the nuclear and mitochondrial data may reflect population bottlenecks followed by population growth that is better captured in rapidly evolving mitochondrial DNA compared with nuclear data.

The Kingston Saltpeter jaguar falls at the base of the older of the two jaguar subclades, which contains two lineages sampled from South America (Jaguar 12 from Guatemala and KM236783.1 from Brazil) and a lineage from North America (Jaguar 11 from Arizona). Given this phylogeny and the estimated age of the common ancestor, it is possible that the two North American jaguars represent lineages that re-colonized North America. The Kingston Saltpeter jaguar lineage may have dispersed northward around the MIS 11 interglacial (~420 to 370 kya), or both lineages could have dispersed northward more recently. Recolonization of Central and North America by lineages from South America has been suggested previously for jaguars (Eizirik et al. 2001; Ruiz-García et al. 2013; Lorenzana et al. 2022), and is the most likely explanation for the present-day distribution of pumas (Saremi et al. 2019). It is also possible, however, that jaguars persisted in North America since their arrival prior to 1 mya but contracted to the southern states toward the end of the Pleistocene, and that the Kingston Saltpeter jaguar is a member of this resident lineage. This alternate hypothesis is supported by the abundance of late Pleistocene jaguar fossils at sites in the southern US compared with more northern regions (Seymour 1993). Regardless, the phylogenetic position of the jaguar sampled from Arizona in our tree (tip 11, Fig. 2, GenBank OR863186) as sister to a South American jaguar (KM236783.1 from Brazil) provides evidence for at least some dispersal between continents during the late Pleistocene. These data also highlight the importance of geographic sampling as, in the absence of data from Brazil and Guatemala, the Kingston Saltpeter and Arizona jaguars would have been inferred as a separate northern lineage.

Today, jaguars are distributed from northern Mexico to central Argentina, with the actual occupied area presenting a >50% reduction within the last century (De La Torre et al. 2017). Our conclusion that the Kingston Saltpeter jaguar falls within the extant diversity of jaguars suggests that this range reduction has been even more drastic since the Holocene. Finally, while our data indicate that the Kingston Saltpeter jaguar does not represent a distinct North American taxon, it is possible that future sampling of older fossils or fossils from additional geographic locations may reveal multiple jaguar lineages within North America. In this way, continued improvements in the recovery of ancient DNA from fossils will further the understanding of taxonomic diversification of lineages during the Pleistocene.

## Supplementary material

Supplementary material is available at Journal of Heredity Journal online.

esad082_suppl_Supplementary_Tables_S1-S2

## Data Availability

The mitochondrial assembly of the Pleistocene jaguar was deposited at NCBI under GenBank accession number OR327006. Mitogenomes from recent jaguars have been deposited at NCBI with accession numbers OR863186 to OR863197.
