# Supplementary material for: Mitogenomic analysis of a late Pleistocene jaguar from North America
Source: J Hered. 2023 Dec 27;115(4):424–31. doi: 10.1093/jhered/esad082 (PMC11235123; doi:10.1093/jhered/esad082)
Supplement: esad082_suppl_Supplementary_Tables_S1-S2 [file esad082_suppl_supplementary_tables_s1-s2.docx]

**Mitogenomic analysis of a late Pleistocene jaguar from North America**

Megha Srigyan^1^, Blaine W. Schubert^2^, Matthew Bushell^2^, Sarah H. D. Santos^3,4^, Henrique Vieira Figueiró^4,5^, Samuel Sacco^1^, Eduardo Eizirik^4^, Beth Shapiro^1,6^

**Supplementary Information**

**Supplementary Table 1. Posterior distribution of the rate variation across lineages (ucld.mean and ucld.stdev) along with estimated clock rates for each partition.**

|  | **Non-coding** | | | **Coding** | | | **D-loop** | | |
| --- | --- | --- | --- | --- | --- | --- | --- | --- | --- |
| **Summary Statistic** | **ucld.mean** | **ucld.stdev** | **mean rate** | **ucld.mean** | **ucld.stdev** | **mean rate** | **ucld.mean** | **ucld.stdev** | **mean rate** |
| **mean** | 2.2E-08 | 1.2E-06 | 1.4E-08 | 3.6E-08 | 2.1E-08 | 3.4E-08 | 1.7E-07 | 8.1E-07 | 5.4E-08 |
| **stderr of mean** | 2.4E-09 | 8.9E-07 | 9.4E-11 | 4.0E-10 | 3.8E-10 | 2.5E-10 | 2.7E-09 | 3.0E-08 | 3.6E-10 |
| **stdev** | 3.8E-08 | 1.8E-05 | 4.1E-09 | 1.2E-08 | 1.3E-08 | 9.7E-09 | 1.0E-07 | 1.3E-06 | 1.7E-08 |
| **median** | 1.4E-08 | 1.5E-08 | 1.3E-08 | 3.4E-08 | 1.8E-08 | 3.3E-08 | 1.5E-07 | 4.4E-07 | 5.2E-08 |
| **95% HPD interval** | [3.5E-9, 5.7E-8] | [1.5E-9, 5.9E-7] | [6.6E-9, 2.2E-8] | [1.4E-8, 6E-8] | [5.3E-9, 4.5E-8] | [1.6E-8, 5.3E-8] | [3.6E-8, 3.7E-7] | [2.9E-8, 2.6E-6] | [2.5E-8, 8.6E-8] |

**Supplementary Table 2** Posterior distribution of substitution rates for different partitions

|  | **Noncoding GTR rates** | | | | | | **Coding GTR rates** | | | | | | **D-loop GTR rates** | | | | | |
| --- | --- | --- | --- | --- | --- | --- | --- | --- | --- | --- | --- | --- | --- | --- | --- | --- | --- | --- |
| **Summary Statistic** | **AC** | **AG** | **AT** | **CG** | **CT** | **GT** | **AC** | **AG** | **AT** | **CG** | **CT** | **GT** | **AC** | **AG** | **AT** | **CG** | **CT** | **GT** |
| **mean** | 0.15 | 2.00 | 0.16 | 0.11 | 3.55 | 0.04 | 0.08 | 3.11 | 0.05 | 0.03 | 2.68 | 0.06 | 0.33 | 2.16 | 0.15 | 0.34 | 2.86 | 0.16 |
| **stderr of mean** | 3e-4 | 2e-3 | 3e-4 | 3e-4 | 2e-3 | 2e-4 | 7e-5 | 1e-3 | 6e-5 | 9e-5 | 1e-3 | 1e-4 | 6e-4 | 2e-3 | 4e-4 | 9e-4 | 2e-3 | 6e-4 |
| **stdev** | 0.04 | 0.19 | 0.04 | 0.05 | 0.19 | 0.03 | 9e-3 | 0.09 | 8e-3 | 0.01 | 0.08 | 0.01 | 0.07 | 0.23 | 0.05 | 0.11 | 0.24 | 0.08 |
| **median** | 0.14 | 1.99 | 0.16 | 0.10 | 3.55 | 0.04 | 0.08 | 3.11 | 0.05 | 0.03 | 2.68 | 0.05 | 0.32 | 2.16 | 0.15 | 0.33 | 2.86 | 0.15 |
| **95% HPD interval** | [0.07, 0.22] | [1.63, 2.35] | [0.09, 0.23] | [0.02, 0.20] | [3.17, 3.92] | [8e-4, 0.1] | [0.06, 0.09] | [2.94, 3.27] | [0.03, 0.06] | [0.01, 0.05] | [2.52, 2.85] | [0.03, 0.08] | [0.20, 0.47] | [1.73, 2.60] | [0.06, 0.24] | [0.14, 0.55] | [2.40, 3.35] | [4e-3, 0.31] |

# 
